# Expanding Genotype–Phenotype Correlation of *CLCNKA* and *CLCNKB* Variants Linked to Hearing Loss

**DOI:** 10.3390/ijms242317077

**Published:** 2023-12-03

**Authors:** Yejin Yun, Sang Soo Park, Soyoung Lee, Heeyoung Seok, Seongyeol Park, Sang-Yeon Lee

**Affiliations:** 1 Department of Otorhinolaryngology-Head and Neck Surgery, Seoul National University Hospital, Seoul National University College of Medicine, Seoul 03080, Republic of Korea; 2 GENOME INSIGHT TECHNOLOGY Inc., Daejeon 34051, Republic of Koreasypark@genomeinsight.net (S.P.); 3 Department of Transdisciplinary Research and Collaboration, Genomics Core Facility, Biomedical Research Institute, Seoul National University Hospital, Seoul 03080, Republic of Korea; 4 Department of Genomic Medicine, Seoul National University Hospital, Seoul 03080, Republic of Korea; 5 Sensory Organ Research Institute, Seoul National University Medical Research Center, Seoul 03080, Republic of Korea

**Keywords:** sensorineural hearing loss, *CLCNKA*, *CLCNKB*, whole-genome sequencing

## Abstract

The ClC-K channels *CLCNKA* and *CLCNKB* are crucial for the transepithelial transport processes required for sufficient urinary concentrations and sensory mechanoelectrical transduction in the cochlea. Loss-of-function alleles in these channels are associated with various clinical phenotypes, ranging from hypokalemic alkalosis to sensorineural hearing loss (SNHL) accompanied by severe renal conditions, i.e., Bartter’s syndrome. Using a stepwise genetic approach encompassing whole-genome sequencing (WGS), we identified one family with compound heterozygous variants in the ClC-K channels, specifically a truncating variant in *CLCNKA* in trans with a contiguous deletion of *CLCNKA* and *CLCNKB*. Breakpoint PCR and Sanger sequencing elucidated the breakpoint junctions derived from WGS, and allele-specific droplet digital PCR confirmed one copy loss of the *CLCNKA*_*CLCNKB* contiguous deletion. The proband that harbors the *CLCNKA_CLCNKB* variants is characterized by SNHL without hypokalemic alkalosis and renal anomalies, suggesting a distinct phenotype in the ClC-K channels in whom SNHL predominantly occurs. These results expanded genotypes and phenotypes associated with ClC-K channels, including the disease entities associated with non-syndromic hearing loss. Repeated identification of deletions across various extents of *CLCNKA_CLCNKB* suggests a mutational hotspot allele, highlighting the need for an in-depth analysis of the *CLCNKA_CLCNKB* intergenic region, especially in undiagnosed SNHL patients with a single hit in *CLCNKA*.

## 1. Introduction

The ClC-K channels, which constitute a subgroup of ClC channels, are expressed in the loop of Henle in the kidneys and stria vascularis of the inner ear [1]. *CLCNKA* and *CLCNKB*, which are both ClC-K channels, play a crucial role in the transepithelial transport processes essential for water resorption and sufficient urinary concentration [2,3,4]. *CLCNKA* is primarily expressed in the thin ascending limb of Henle and is involved in passive NaCl transport [3]. By contrast, *CLCNKB*, which is primarily expressed in the thick ascending limb of Henle, contributes to transcellular Cl^−^ reabsorption and ion exchange balance in cooperation with NKCC1 and Na^+^/K^+^-ATPase [5]. *CLCNKB* is essential for sodium chloride reabsorption in the kidney’s outer medulla and cortical region [5].

Additionally, *CLCNKA* and *CLCNKB* are expressed in basolateral membranes of marginal cells of the cochlear stria vascularis, where they play a role in the secretion of K^+^ into the endolymph via NKCC1 and Na^+^/K^+^-ATPase. The stria vascularis is responsible for creating the high K^+^ concentration of 140 mM and the positive potential of +100 mV in the endolymph that fills the scala media [6]. It is involved in K^+^ recycling within the cochlea and maintaining the balance of chloride, salt, and water in both the scala media and the stria vascularis. Understanding the transport processes needed to uphold this balance is crucial [7]. This balance, in turn, is essential for sensory mechanoelectrical transduction (MET) in the cochlear hair cells.

Loss-of-function alleles in ClC-K channels and barttin (a beta-subunit of ClC-K channels) are associated with a range of clinical phenotypes, including Bartter syndrome (BS) [1,8,9]. BS is a heterogenous genetic disorder marked by diverse symptoms such as impaired urinary concentration, excessive urine production, increased thirst, low potassium levels, alkalosis, high levels of plasma renin and aldosterone, and normal blood pressure [1]. Clinically, five distinct types of BS are recognized, each associated with a specific disease-causing gene [10]. Specifically, *CLCNKB* was identified as an underlying disease gene for type III BS [11]. Moreover, the presence of two loss-of-function alleles in both ClC-K channels (*CLCNKA* and *CLCNKB*), as a digenic trait, leads to severe renal salt wasting and sensorineural deafness, which is classified as type IV BS [12]. This is consistent with the severe phenotypes caused by variants in *BSND*, which encodes barttin [13], despite some variants (e.g., p.Ile12Thr and p.Val33Leu) leading to DFNB73 [14,15].

Although the exact mechanism remains poorly understood, dysfunction in either ClC-K or barttin channels can hinder the countercurrent mechanism in the loop of Henle, thereby affecting water reabsorption into the collecting duct [1]. Additionally, K^+^ recycling, coupled with maintaining the balance of chloride, salt, and water, in both the scala media and the stria vascularis are crucial. The inner-ear-specific disruption of Bsnd in mice led to a reduced driving force for potassium entry through MET channels into sensory hair cells, resulting in the degeneration of the organ of corti and profound hearing loss [6]. Consequently, impaired ClC-K/barttin channels can disturb cochlear ion balance, potentially disrupting endocochlear potential and leading to SNHL in human BS [6].

While the expression of ClC-K channels in the stria vascularis and their role in hearing have been characterized, hearing loss phenotypes resulting from defects in these ClC-K channels and their associated genotype–phenotype correlations remain poorly understood. This study presents the first report of the non-syndromic hearing loss caused by compound heterozygous variants, specifically a truncating variant in *CLCNKA* in trans with contiguous deletion of *CLCNKA* and *CLCNKB*, and refines the genotype–phenotype correlations in the context of ClC-K channels. Expanding our knowledge of phenotypes associated with ClC-K channels beyond BS aids in predicting the natural course of these conditions, ending the diagnostic odyssey, and applying suitable clinical management strategies [16,17].

## 2. Results

### 2.1. Phenotype

**Figure 1:**
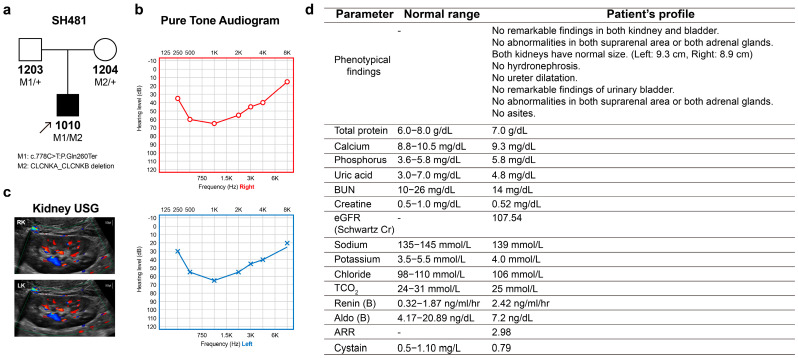
Pedigree and clinical phenotypes. (**a**) Pedigree of family SH481. (**b**) Audiological phenotypes of the proband, exhibiting symmetric, moderately severe, and cookie-bite configuration. (**c**,**d**) The sonography findings were unremarkable in the kidneys, bladder, suprarenal areas, and adrenal glands (RK, right kidney; LK, left kidney). Laboratory data of the proband regarding renal function. gram g; milligram mg; nanogram ng; deciliter dL; milliliter mL; hour h; ultrasonography USG.

In a cohort of 428 SNHL probands who underwent genetic testing, one proband (SH481-1010) was found to harbor compound heterozygous variants, specifically a truncating variant in *CLCNKA* in trans with a contiguous deletion of *CLCNKA* and *CLCNKB*. The genetic contribution of ClC-K channels to SNHL has been identified to be approximately 0.23%. The proband (male, 9 years old) was a sporadic case showing signs of hearing impairment with post-lingual onset (Figure 1a). In this family (SH481), there was no history of hearing loss across three generations. The proband passed the newborn hearing screening test at birth. A review of the proband’s medical history and physical examinations revealed no significant findings or underlying diseases. The proband’s overall development was within the normal range. Specifically, the weight, height and head circumference of the proband were within the normal range, aligning with the normal growth curve. However, near the age of 6–7, the proband began to exhibit signs of hearing impairment, characterized by the frequent repetition of questions within an elementary school environment. The audiogram of the proband revealed bilaterally symmetric moderate-to-severe SNHL with a characteristic “cookie-bite” configuration (Figure 1b). The audiograms, which were closely monitored over a two-year period, displayed no evidence of progressive deterioration in hearing loss. Radiologic imaging did not detect any inner ear anomalies or brain lesions. A review of the proband’s medical history and physical examinations revealed no significant findings or underlying diseases. The proband’s overall development was within the normal range. After the genetic diagnosis, the proband was referred to pediatric nephrologists for the evaluation of renal involvement such as BS. Kidney sonography showed both kidneys to be of normal size (left: 9.3 cm, right: 8.9 cm), with no abnormalities in either kidney or bladder (Figure 1c). Additionally, laboratory tests were also found to be normal, without evidence of hypokalemic alkalosis (Figure 1d). Consistent with this, the urinary analysis showed normal range (Table S1), precluding the latent signs of loop dysfunction

### 2.2. Exome Sequencing and Data Analysis

Exome sequencing and bioinformatic analysis were used to prioritize candidate variants for hearing loss (Table S2). This strategy resulted in the identification of four heterozygous variants, *MPZL2* c.220C>T:p.Gln74*, *NOTCH1* c.1945C>T:p.Pro649Ser, APAF1 c.2887C>T:p.Gln963*, and *CLCNKA* c.778C>T:p.Gln206*, which were inconclusive at this stage. The *CLCNKA* heterozygous nonsense variant has been reported previously [12], and the variant was from the maternal allele (Figure S2). The *CLCNKA* p.Gln206* is considered “pathogenic” based on the American College of Medical Genetics and Genomics/Association for Molecular Pathology (ACMG/AMP) guidelines [18].

### 2.3. Whole Genome Sequencing and Data Analysis

**Figure 2:**
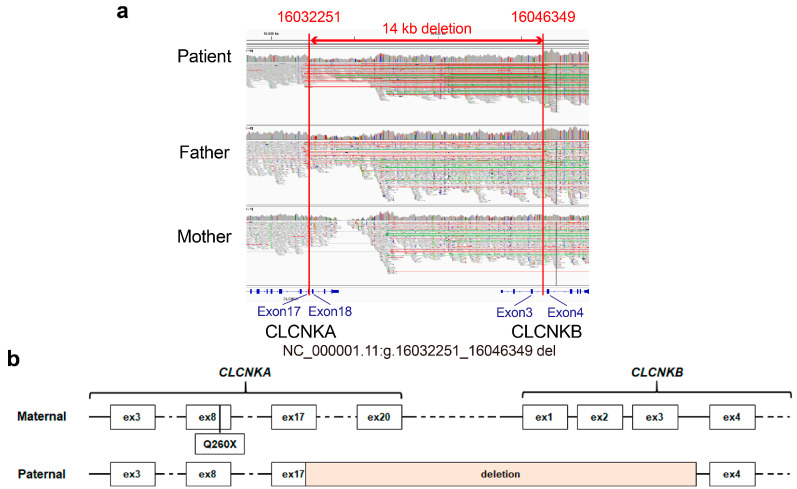
IGV view of the 14 kb contiguous deletion and *CLCNKA* variant derived from parent. (**a**) The IGV revealed a large contiguous deletion in *CLCNKA* and *CLCNKB* spanning g.16032251 to g.16046349, with the 5′-breakpoint at exon 17 of *CLCNKA* and 3′-breakpoint at intron 3 of *CLCNKB* being inherited from the father. (**b**) Schematic illustration of biallelic variants of ClC-K channels.

To investigate whether there were regions and genetic variants not technically covered by exome sequencing, such as structure variations (SVs) and genomic rearrangements, familiar WGS, which included the patient’s father and mother, was performed. Importantly, a heterozygous contiguous *CLCNKA* and *CLCNKB* deletion spanning g.16032251 to g.16046349 (the 5′-breakpoint at exon 17 of *CLCNKA* and the 3′-breakpoint at intron 3 of *CLCNKB*) with 1 bp of microhomology was identified (Figure 2a). Resultantly, the proband harbored compound heterozygous variants, specifically a truncating variant in *CLCNKA* in trans with a contiguous deletion of *CLCNKA* and *CLCNKB*, the lack of homology in the identified breakpoints suggests that the deletion resulted from non-homologous end joining, not homologous recombination. In the gnomAD SV v2.1 dataset, a deletion involving *CLCNKA_CLCNKB* (variant ID: DEL_1_1318) was reported with an allele frequency of 2.75 × 10^−4^. However, the dataset, which encompasses 10,847 samples, did not document any identical structural variation reported herein.

### 2.4. Molecular Genetic Analysis

**Figure 3:**
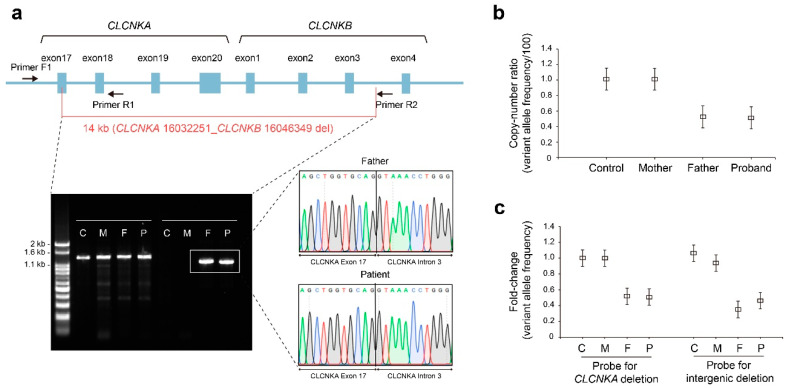
Validation of breakpoint junctions and copy number of large deletion region. (**a**) Confirmation of breakpoint junctions of a large heterozygous contiguous deletion involving *CLCNKA* and *CLCNKB*. The large contiguous deletion was verified using a forward primer located within intron 15 of *CLCNKA* (Primer F1) and a reverse primer located within intron 18 of *CLCNKA* (Primer R1) and intron 3 of *CLCNKB* (Primer R2). The amplicon produced by primers F1 and R1 was 1326 bp, while that produced by primers F1 and R2 was 1222 bp; these were detected in the proband and his father. Direct Sanger sequencing revealed the deletion breakpoints, consistent with the genomic positions in WGS. The original gel image is included in Figure S1. (**b**) The variant allele frequency of *CLCNKA* via ddPCR. (**c**) Fold-changes of the variant allele frequency with *CLCNKA* and the intergenic region between *CLCNKA* and *CLCNKB*. C, control; M, mother; F, father; P, proband.

To elucidate the breakpoint junctions derived from WGS, a forward primer at intron 15 of *CLCNKA* (Primer F1) and a pair of reverse primers (Primer R1 at intron 18 of *CLCNKA* and Primer R2 at intron 3 of *CLCNKB*) were designed. The breakpoints detected by WGS were verified by amplifying the 1222 bp product using primers F1 and R2 across the 5′ and 3′ breakpoints; the 1222 bp amplicon was observed in the proband and father, but not in the control or mother (Figure 3a). We then performed digital droplet PCR (ddPCR) to confirm the WGS observations. The variant allele frequency (VAF) was calculated from the fraction of total droplets containing a target and deletion-specific positive droplets, based on the Poisson distribution and fitting algorithm (Figure S3). In the region of *CLCNKA*, the VAF proportions with normal control and mother were 101.3% and 101.1%, respectively, while the father’s and patient’s proportions were 52.4% and 51.3%, respectively (Figure 3b). The VAF fold-change of both the *CLCNKA* and intergenic regions showed a similar pattern (Figure 3c). Accordingly, allele-specific droplet digital PCR confirmed one copy loss of the *CLCNKA*_*CLCNKB* contiguous deletion.

### 2.5. Phenotype–Genotype Correlation

**Figure 4:**
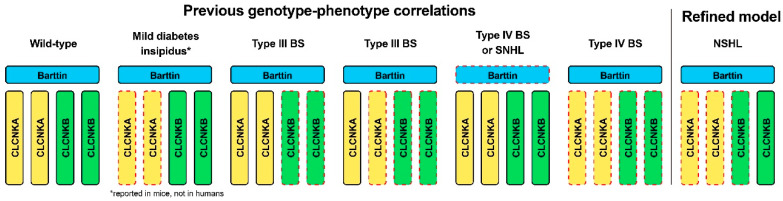
Schematic illustration of genotype–phenotype correlations in relation to variants in ClC-K channels. The red dotted lines indicate the genotypes (i.e., mutant allele state) corresponding to the defects in each channel. While previously reporting genotype–phenotype correlations, the current study proposed a refined model characterized specifically by non-syndromic hearing loss. *, mild diabetes insipidus phenotype, caused by the null alleles of *CLCNKA* but normal *CLCNKB* and *BSND* alleles, has been reported in mice, not in humans. BS, Bartter syndrome; NSHL, non-syndromic hearing loss.

Previous studies reported that biallelic variants in *CLCNKA* lead to a mild form of diabetes insipidus in knockout mice [19], but not in humans. In contrast, either biallelic variants in *CLCNKB* or compound heterozygous for a truncating variant in *CLCNKB* in trans with a heterozygous contiguous deletion in *CLCNKA* and *CLCNKB* are associated with type III BS. In addition, compound or digenic variants of both *CLCNKA* and *CLCNKB* channels or variants in the accessory subunit barttin have been linked to type IV BS. In the literature review, among the spectrum of clinical phenotypes reported for variants of the *CLCNKA* and *CLCNKB*, those associated with hearing loss are primarily linked with Type III or Type IV BS (Table 1). In this context, the case defines a distinct subset of phenotypes for variants in *CLCNKA* and *CLCNKB* channels in whom renal phenotypes are absent. Specifically, a heterozygous truncating variant in *CLCNKA* in trans, along with a heterozygous contiguous deletion in *CLCNKA* and *CLCNKB*, resulted in non-syndromic hearing loss. Accordingly, we propose a refined phenotype–genotype correlation in the context of ClC-K channels, expanding disease entities associated with non-syndromic hearing loss (Figure 4).

## 3. Discussion

In this study, we report the first identification of non-syndromic hearing loss caused by compound heterozygous variants, specifically a truncating variant in *CLCNKA* in trans with a contiguous deletion of *CLCNKA* and *CLCNKB*. Our results support a DFNB96 linkage region at 1p36.31-p36.1 associated with autosomal recessive non-syndromic hearing loss, which includes the *CLCNKA* and *CLCNKB* [21]. In the literature, there have been three cases that presented hearing loss attributed to *CLCNKA* and *CLCNKB* variants (see Table 1). However, detailed documentation of the hearing loss phenotype was available for only one case harboring a homozygous mutation in *CLCNKB* (c.229G>C:p. Ala77Pro). In the previously reported case, the patient displayed bilateral moderately severe SNHL with a flat configuration (250 Hz to 9 kHz). Conversely, our case showed bilateral moderately severe SNHL with a cookie-bite audiogram configuration. To elucidate our understanding of such audiological phenotypes in BS, access to numerous variants of the implicated gene and profound audiologic data are essential. Therefore, as presented herein, a detailed analysis of both the audiological data and the genotypes in these rare instances is imperative. With this case, we expand the genotypes and phenotypes associated with ClC-K channels and refine the genotype–phenotype correlation in the context of ClC-K channels, expanding our understanding of the disease entities associated with non-syndromic hearing loss.

The stepwise genetic testing pipeline used in this study, which combines trio-based WGS with breakpoint PCR and Sanger sequencing, is an effective approach for deciphering a contiguous *CLCNKA*_*CLCNKB* deletion in undiagnosed cases, even after exome sequencing. In this study, WGS precisely indicates the 5- and 3-end breakpoints, and the breakpoints were accurately validated by breakpoint PCR and Sanger sequencing. In accordance with this approach, the breakpoints of cryptic genomic copy number variations (CNVs) identified through WGS were reassessed using Sanger sequencing or breakpoint PCR, following a stepwise methodology [22]. In this study, the allele-specific ddPCR further confirmed a heterozygous contiguous deletion of *CLCNKA* and *CLCNKB*. In the literature, there is a lack of detection schemes for the effective identification of cryptic genomic CNVs. Our study suggests that the application of stepwise genomic pipelines could offer a framework for the detection and confirmation of the SVs, including those with a heterozygous contiguous *CLCNKA* and *CLCNKB* deletion.

The large *CLCNKA*_*CLCNKB* deletion has been previously detected in patients with type III and IV BS [6,9,23], suggesting a mutational hotspot allele. Indeed, a *CLCNKA*_*CLCNKB* deletion was reported in the Genome Aggregation Database (gnomAD) SV v2.1 (DEL_1_1318) dataset with an allele frequency of 2.8 × 10^−4^, albeit with deletions across various extents of *CLCNKA_CLCNKB*. The deletion likely occurs through non-allelic homologous recombination-mediated deletion, since the two breakpoints exhibit high homology [24]. The progress in genomic technologies, coupled with reduced sequencing costs and improved data management, has made extraordinary strides toward deciphering the complex genetic architecture and associated mechanisms underlying human genetic disorders. In parallel with this, our observation highlights the importance of in-depth analysis for the *CLCNKA_CLCNKB* deletion in undiagnosed patients with SNHL, especially when one pathogenic allele in the *CLCNKA* is present.

Exome sequencing is effective in identifying point mutations in the coding region. However, it has a limitation in detecting most SVs as the breakpoints of SVs are mostly located in non-coding regions. Although copy-number changes in exome sequencing can be used to infer deletions, it can be challenging to draw conclusions, especially in homologous regions like *CLCNKA* and *CLCNKB*. WGS theoretically has a higher ability to identify a more diverse spectrum of variant profiles, including deep intronic, regulatory, andSVs, compared to exome sequencing [25]. Accordingly, its coverage includes the findings of all other genomic approaches. Moreover, a previous study also demonstrated the clinical utility of WGS to establish the correct diagnosis in renal tubular disorders with overlapping phenotypes [26]. Given this, WGS can also be considered as a first-line diagnostic option, especially in rare diseases with genetic heterogeneity and a non-specific phenotype [27]. Nevertheless, WGS has not been widely applied in human genetic disorders as a first-line diagnostic tool, primarily due to challenges associated with the difficulties of bioinformatic analysis and precise clinical interpretation. As proposed herein, a stepwise genomic approach, exome sequencing followed by WGS, could offer an effective clinical guideline for real-world SNHL practice. 

ClC channels and transporters, expressed across a wide range of tissues, fulfill diverse functions in paly. Specifically, *CLCNKA* and *CLCNKB*, which are part of the human ClC channel family, facilitate both inward and outward currents. This dual functionality supports transcellular chloride fluxes in numerous tissues, notably in the inner ear and kidney [28]. The type III BS, which is characterized by hypokalemic metabolic alkalosis without significant urinary calcium excretion or hearing loss, is attributed to compound heterozygous variants with a heterozygous nonsense variant in *CLCNKB* in addition to this large deletion [11]. In comparison, as observed herein, non-syndromic hearing loss is attributed to compound heterozygous variants with a nonsense *CLCNKA* variant in trans, with the large deletion spanning *CLCNKA* and *CLCNKB*. The distinct presentation of non-syndromic hearing loss in the case involving a single copy loss of *CLCNKB* along with biallelic loss-of-function alleles in *CLCNKA* might be understood through the differential roles that these genes have in the inner ear and kidney. Perhaps, *CLCNKA* appears to be less susceptible in terms of maintaining the ion balance necessary for proper signal transduction in inner hair cells when mutated. When compounded with heterozygous variants of *CLCNKB*, the dysfunction of *CLCNKA* can eventually disrupt the ion transport balance necessary for maintaining the endocochlear potential, leading to SNHL. Conversely, while *CLCNKB* is also involved in ion transport in the inner ear, its impact on hearing might be less pronounced than *CLCNKA*. This is evident from clinical observations where *CLCNKB* mutations predominantly affect renal function with minimal or no impact on hearing (i.e., BS type III). Alternatively, this is likely due to the presence of other compensatory mechanisms in the kidney and inner ear. Similarly, one *BSND* variant (p.Ile12Thr and p.Val33Leu) causes non-syndromic hearing loss (DFNB73) without affecting renal function, which supports the differential susceptibility of inner ear and kidney function to the dysfunction of the CIC-K/barttin channels [1,15].

There have been no reports on human diseases caused by variants solely in *CLCNKA*. However, research on *CLCNKA* knockout mice has revealed the presence of a mild form of diabetes insipidus [19]. This milder phenotype in *CLCNKA* mutants can be attributed to compensatory mechanisms between the two homologous ClC-K channels. The current case suggests that an additional copy gene defect in *CLCNKB*, in combination with biallelic loss-of-function alleles in *CLCNKA*, contributed to the development of the hearing loss phenotype. The expression of ClC-K channels in the inner ear is crucial for maintaining proper hearing function [1], and the co-expression of barttin, an accessory subunit of ClC-K channels, led to plasma membrane localization and improved stability of these channels via protein–protein interaction [29]. Given that our WGS did not reveal any variants in *BSND*, it is possible that the additional *CLCNKB* single-copy defect disrupts protein stability and reduces the membrane expression level below the reference threshold necessary for maintaining inner ear function, despite the compensatory roles of the ClC-K channels. The mechanism through which additional *CLCNKB* single-copy defects result in hearing loss in cases where *CLCNKA* is non-functional remains to be elucidated.

At this point, the precise mechanism underlying the non-syndromic hearing loss caused by compound heterozygous variants, specifically a truncating variant in *CLCNKA* in trans with a contiguous deletion of *CLCNKA* and *CLCNKB*, remains elusive. The limitation stemming from reporting on a single family, despite ongoing efforts to recruit additional families via gene-matcher [30], cannot be overlooked. The application of CRISPR-engineered cellular and organism models may prove this finding by exploring the influence of individual gene loci, thus aiding in the study of multigenic human traits. Furthermore, it would be essential to move beyond exploring the target gene’s function and instead examine its broader contribution within the molecular networks and polygenic risk scores. Previous research has indicated a potential link between *CLCNKB* polymorphisms and conditions such as hypertension and reduced hearing thresholds [31,32]. On the other hand, polymorphisms in *CLCNKA* have been associated with elevated arterial pressure [33]. Despite these associations, the functional consequences of these polymorphisms in relation to BS clinical phenotypes have not been fully explored.

Nevertheless, these findings contribute to the growing body of knowledge regarding genotype–phenotype correlations in the context of ClC-K channels, enhancing our understanding of the disease entity associated with non-syndromic hearing loss. Moreover, our observations suggest the significance of the in-depth genomic analysis of the *CLCNKA*_*CLCNKB* deletion in patients with SNHL who remain undiagnosed, particularly when one pathogenic allele in *CLCNKA* is identified.

## 4. Materials and Methods

### 4.1. Participants and Clinical Assessment

All procedures in this study were approved by the Institutional Review Boards of Seoul National University Hospital (IRB-H-0905-041-281). This study utilized a retrospective design and focused on participants attending the Hereditary Hearing Loss Clinic within the Otorhinolaryngology division at the Center for Rare Diseases, Seoul National University Hospital, Korea, from March 2021 to May 2023. Of total, we decomposed 428 unrelated SNHL families who underwent a stepwise approach encompassing exome sequencing and WGS. The demographics of 428 probands were summarized in Table S3. The clinical profiles were retrieved from electronic medical chart, including medical history interviews, physical examinations, imaging, and audiological assessments.

### 4.2. Exome Sequencing and Bioinformatic Analysis

Genomic DNA was extracted from peripheral blood samples utilizing the Chemagic 360 instrument (Perkin Elmer, Baesweiler, Germany). The exome sequencing was performed through SureSelectXT Human All Exon V5 (Agilent Technologies, Santa Clara, CA, USA). We prepared a library that was paired-end sequenced using a NovaSeq 6000 system (Illumina, San Diego, CA, USA) with an average coverage depth of 100×. The sequence reads were aligned to the human reference genome (GRCh38) and processed following the guidelines of the Genome Analysis Toolkit (GATK) [34] to identify single nucleotide variations (SNVs) and indels. As previously described [35], a comprehensive bioinformatic approach was carried out to find candidate variants using a distinct filtering process. We applied the ANNOVAR software [36] for variant annotation. As previously described [37,38,39], bioinformatics analysis and strict filtering were performed to retrieve candidate variants of hearing loss: (i) Non-synonymous variants with quality scores > 30 and read depths > 10 were selected. (ii) All variants with minor allele frequencies (MAFs) ≤ 0.001 were chosen based on population database, including the gnomAD (https://gnomad.broadinstitute.org/) (accessed on 1 May 2023), and ethnically-matched controls, including Korean Variant Archive, for a reference database of genetic variations in the Korean population (KOVA2) (https://www.kobic.re.kr/kova/search_detail_view_hg19) (accessed on 1 May 2023). (iii) The pathogenic potential of each variant was determined using in silico tools (Combined Annotation Dependent Depletion (CADD), https://cadd.gs.washington.edu/ and Rare Exome Variant Ensemble Learner (REVEL), https://sites.google.com/site/revelgenomics/) (accessed on 1 May 2023). In addition, we used the GERP ++ score from the UCSC Genome Browser (http://genome.ucsc.edu/) (accessed on 1 May 2023) to estimate the evolutionary conservation of the amino acid sequences. (iv) Furthermore, compatibility with inheritance patterns and audiological/clinical phenotypes was evaluated. Additionally, the ClinVar and HGMD databases were screened to check whether candidate variants had been previously identified in other patients. (v) The candidate variants were confirmed through Sanger sequencing, and a segregation study was performed using parental DNA samples. Resultantly, we classified the pathogenicity of candidate variants according to the ACMG-AMP guidelines for SNHL [40].

### 4.3. Whole-Genome Sequencing and Bioinformatic Analysis

DNA libraries for WGS were generated using the TruSeq DNA PCR-Free kit (Illumina, San Diego, CA, USA) from 1 µg of genomic DNA. WGS was conducted on the NovaSeq platform 6000 (Illumina) to generate 151 bp paired-end reads. The raw sequencing data analysis and downstream interpretation were performed using RareVision^TM^ (Genome Insight, Inc., Daejeon, Republic of Korea). The sequenced reads were mapped to the GRCh38 using the BWA-MEME algorithm, and duplicated reads were removed using SAMBLASTER [41]. Mean coverages of samples were 31.16, 27.16, and 33.38 for proband, father, and mother, respectively. Base substitutions and short indels were identified using HaplotypeCaller [34] and Strelka2 [42]. Genomic rearrangements were identified using Delly [43], and the breakpoints of genomic rearrangements of interest were inspected visually and confirmed using the Integrative Genomics Viewer (IGV).

### 4.4. Breakpoint PCR, Digital Droplet PCR, and Sanger Sequencing

Molecular analysis of the genomic rearrangement derived from WGS was conducted using breakpoint PCR and ddPCR. The primers were used for breakpoint PCR (Table S4), and probe and primers were used for ddPCR (Table S5). Genomic DNA extracted from patients were used to perform ddPCR with the QX200^TM^ ddPCR system. Procedures followed the manufacturer’s protocol with minor modifications. Briefly, the master mix for ddPCR consisted of 100 ng of genomic DNA, 1×ddPCR supermix for probes (no dUTP; 186-3023, Bio-Rad, Hercules, CA, USA), 1.0 μM primer, and 0.25 μM probe (metabion, Planegg, Germany). The samples were thoroughly combined before being put into a Bio-Rad QX100^TM^/QX200 droplet generator’s DG8 cartridge. The cartridge was then put into the QX200 Droplet Generator^TM^ (Bio-Rad) and added Droplet Generation Oil. After droplet formation, the droplets were carefully moved to a twin-tec semi-skirted 96-well PCR plate (EP0030128575, Eppendorf, Taufkirchen, Germany). PCR was then carried out in a C1000 Touch thermal cycler (Bio-Rad) for the two-step running program of 94 °C for 30 s and 56 °C for 1 min for 40 cycles with initial and final incubation at 95 °C and 98 °C, respectively. Signal in each droplet was read by a droplet reader and analyzed using QuantaSoft program (Version 1.7) (Bio-Rad). For breakpoint PCR, primers were designed (Table S4) to amplify the suspected breakpoints of deletion identified in *CLCNKA* and *CLCNKB*. The segregation study using Sanger sequencing was performed using parental DNA samples.

### 4.5. Laboratory Tests

A total of 5.0 mL of the patient’s peripheral blood sample was collected in a serum separator tube (SST) for analysis of a renal panel, which included measurements of Calcium, Phosphorus, Uric Acid, Blood Urea Nitrogen (BUN), Creatinine, Sodium, Potassium, Chloride, and Total Carbon Dioxide (TCO_2_). Additionally, Cystatin C levels were evaluated using immunoturbidimetric methods. Furthermore, patient’s peripheral blood sample was also collected in a gel EDTA mixed tube for the assessment of renin activity-basal and aldosterone-basal. Reference values were documented based on age and gender.

## 5. Conclusions

This study presents the first report of non-syndromic hearing loss caused by compound heterozygous variants, specifically a truncating variant in *CLCNKA* in trans with a contiguous deletion of *CLCNKA* and *CLCNKB*, through a stepwise genomic approach encompassing WGS. These results provide an additional basis of genotype–phenotype correlations in the context of ClC-K channels, expanding our understanding of the disease entities associated with non-syndromic hearing loss.

## Figures and Tables

**Table 1:** Literature review with regard to *CLCNKA* and *CLCNKB* variants linked to hearing loss. CLCNKA Refseq transcript accession number NM_005070.4; Refseq protein accession number NP_004061.3. CLCNKB Refseq transcript accession number NM_000085.5; Refseq protein accession number NP_000076.2.

References	Gene	Variant	Zygosity	Age	Clinical Phenotypes		
Robitaille et al., 2011 [20]	*CLCNKB*	c.229G>C:p. Ala77Pro	Homozygote	13-year-old	Batter syndrome type III	Renal phenotype	Severe dehydration secondary to a prolonged diarrhea Chronic metabolic alkalosis Normal renal function
						Hearing phenotype	Sensorineural deafness with a loss of 60–70 dB from 250 to 9 kHz in both ears
Nozu et al., 2008 [12]	*CLCNKA*	c.778C>T:p.Gln260Ter	Heterozygote	2-year-old	Batter syndrome type IV	Renal phenotype	Polyuria and severe volume depletion Hyponatremia/normal potassium concentration Severe hypokalemia and metabolic alkalosis Acute renal failure.
		Deletion (*CLCNKA* exon16- *CLCNKB* intron2)	Heterozygote				
	*CLCNKB*	IVS17+1G>A: Splicing variant	Heterozygote			Hearing phenotype	Bilateral sensorineural deafness
		Deletion (*CLCNKA* exon16- *CLCNKB* intron2)	Heterozygote				
Schlingmann et al., 2004 [9]	*CLCNKA*	c.240G>C:p.Trp80Cys	Homozygote	2-month-old	Batter syndrome type IV	Renal phenotype	Polyuria and volume depletion associated with hypokalemia and metabolic alkalosis
	*CLCNKB*	Whole gene deletion	Homozygote			Hearing phenotype	Bilateral sensorineural deafness

## Data Availability

The variants included this study were submitted to LOVD (https://databases.lovd.nl/shared/individuals/00430364) (accessed on 18 October 2023). All data are available upon request to the corresponding author (Sang-Yeon Lee).

## Supplementary Materials

The supporting information can be downloaded at: https://www.mdpi.com/article/10.3390/ijms242317077/s1.

## Abbreviations

SNHL	Sensorineural hearing loss
WGS	Whole genome sequencing
MET	Sensory mechanoelectrical transduction
BS	Bartter syndrome
ACMG-AMP	American College of Medical Genetics and Genomics/Association for Molecular Pathology
SVs	Structure variations
ddPCR	Digital-droplet PCR
VAF	Variant allele frequency
CNV	Copy number variations
gnomAD	Gemone Aggregation Database
GRCh38	Human reference genome
GATK	Genome analysis toolkit
SNV	Single nucleotide variations
MAFs	Minor allele frequencies
KOVA2	Korean Variant Archive for a reference database of genetic variations in the Korean population
CADD	Combined Annotation Dependent Depletion
REVEL	Rare Exome Variant Ensemble Learner
IGV	Integrative genomics viewer
USG	Ultrasonography
