# Supplementary material for: Expanding Genotype–Phenotype Correlation of CLCNKA and CLCNKB Variants Linked to Hearing Loss
Source: Int J Mol Sci. 2023 Dec 3;24(23):17077. doi: 10.3390/ijms242317077 (PMC10707517; doi:10.3390/ijms242317077)

Supplementary Materials

Supplementary Figure

Figure S1. The original gel images in Figure 3a

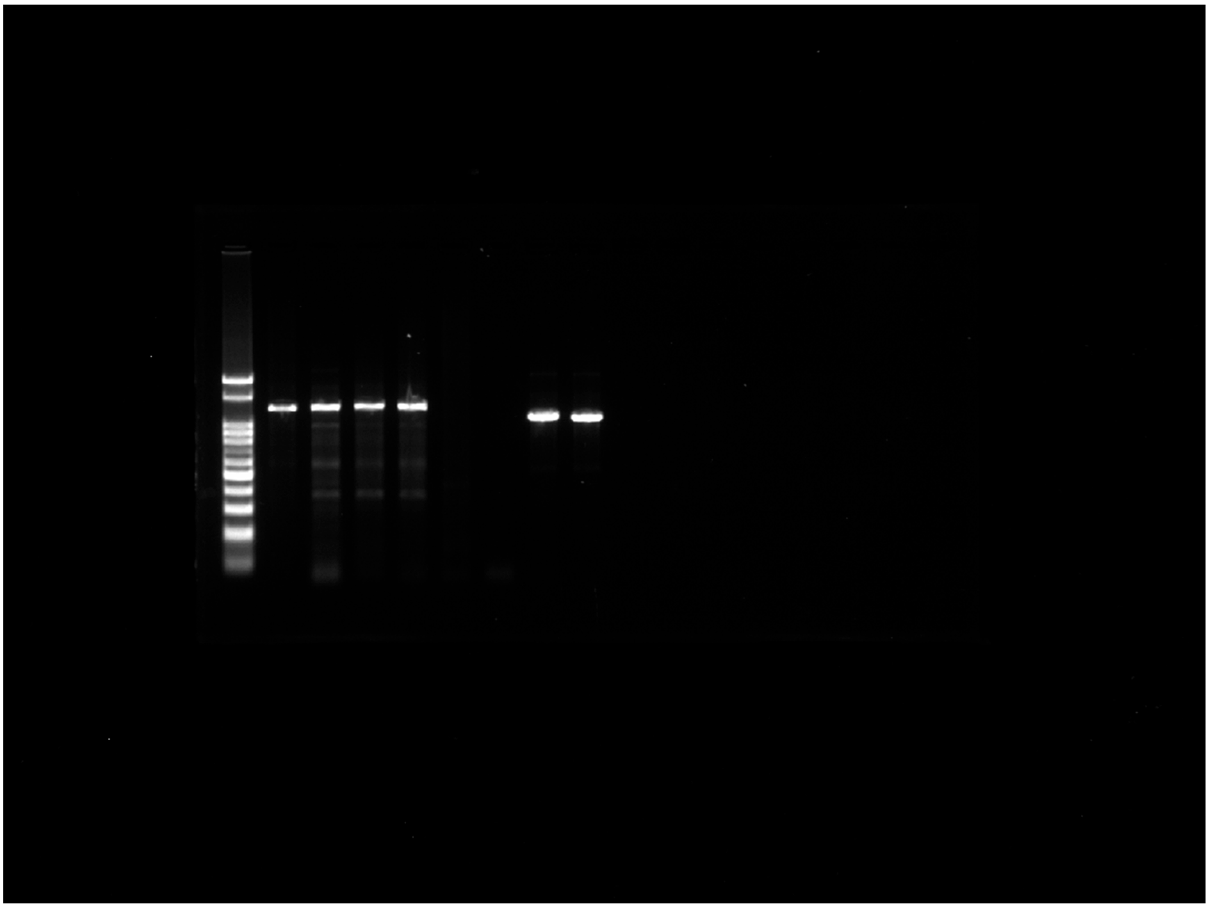

Supplementary Tables

Table S1. Laboratory Data: Urine Analysis of the Proband

Parameter	Patient's profile
**SG**	1.019
**pH**	5.5
**ALB**	-
**GLU -**	
**KET**	-
**BIL**	-
**BLD**	-
**URO**	+/-
**NIT**	-
**WBC(s)**	-
**RBC**	< 1 (1.1/μℓ)
**WBC**	< 1 (1.3/μℓ)
**Squamous cell**	< 1 (0.0/μℓ)
**Transitional cell -**	
**Renal tubular cell -**	
**Bacteria -**	
**Casts -**	
**Crystals -**	
**Yeast -**	
**Sperm -**	
**Others -**	

Table S2. In-silico analysis of candidate variants derived from exome sequencing

Gene	Genomic Position: Change (GRCh37/hg19)	HGVS		In silico Predictions		Alternative Allele Frequency			Clinvar
		Nucleotide change	Amino Acid change	CADD Phred	REVEL	KOVA (5,305 Individuals)	KRGDB (1,722 individuals)	gnomAD	Classification
MPZL2	Chr11:118133802AG-A	c.68delC	p.Pro23Leufs2Ter	NA	NA	ND	ND	Exome (3.993e-06) Genome (ND)	Pathogenic / Likely pathogenic
NOTCH1	Chr9:139409811G-A	c.1945C>T	p.Pro649Ser	27.3	0.795	ND	ND	Exome (ND) Genome (ND)	Uncertain Significance
APAF1	Chr12:99106142C-T	c.2887C>T	p.Gln963Ter	43.0	NA	ND	ND	Exome (ND) Genome (ND)	ND
CLCNKA	Chr1:16027432C-T	c.778C>T	p.Gln260Ter	37.0	NA	ND	0.00290867	Exome (4.395e-05) Genome (ND)	Pathogenic (PMID:18310267)

Abbreviations: Het, heterozygote; VUS, variant uncertain significance; NA, not available; ND, no data available

Sequence Variant Nomenclature (https://mutalyzer.nl/)

HGVS: Human Genome Variation Society (https://www.hgvs.org/)

CADD: Combined Annotation Dependent Depletion (https://cadd.gs.washington.edu/)

REVEL: Rare Exome Variant Ensemble Learner (https://sites.google.com/site/revelgenomics/)

KOVA: Korean Variant Archive for a reference database of genetic variations in the Korean population (http://kobic.re.kr/kova/)

KRGDB: Korean Reference Genome Database (http://152.99.75.168:9090/KRGDB/welcome.jsp)

gnomAD: The Genome Aggregation Database (https://gnomad.broadinstitute.org/)

Table S3. Demographics of 428 unrelated SNHL cohort[a]

	Count(n=428)[b]
**Age**	
0~5	128(29.90)
6~9	48(11.21)
10~19	63(14.72)
20~29	40(9.35)
30~39	41(9.58)
40~49	38(8.88)
50~59	33(7.71)
60~69	29(6.78)
70~79	8(1.87)
**Sex**	
Male	198(46.26)
**Syndromic**	
Apparent Syndromic	41(9.58)
**Hearing Loss Type (Right)**	
Sensorineural	407(95.09)
Mixed	21(4.91)
**Hearing Loss Type (Left)**	
Sensorineural	403(94.16)
Mixed	25(5.84)

[a]Patients with Sensorineural or mixed hearing loss who underwent genetic tests were included.

[b]Data are presented as number (percentage) of patients in our study cohort.

Table S4. Experimental information of breakpoint PCR

Primer	Sequence
**Primer F1**	5'- AAGACCCAAGTGAAGCTGGTCTGGGGGACATG -3'
**Primer R1**	5'- TCCACTGTCTTTCTCCACCCAGAGACTTCCA -3'
**Primer R2**	5'- AGCCACATGACTCTTCCTGGGCCTTTGTCTG -3'

Step	Temp	Time
**Step 1**	95℃	10min
**Step 2**	95℃	30sec
**Step 3**	60℃	30sec
**Step 4**	72℃	90sec (go to Step 2, 40times)
**Step 5**	72℃	5min
**Step 6**	4℃	∞

Abbreviation: minute min; second sec;

We performed PCR using primer designed and conducted at the condition indicated at table S1. Primer F1 (forward primer) combines *CLCNKA* exon 17, sequence is 5'- AAGACCCAAGTGAAGCTGGTCTGGGGGACATG -3'. Primer R1 (reverse primer) combines CLCNKA intron 17, sequence is 5'- TCCACTGTCTTTCTCCACCCAGAGACTTCCA -3'. Primer R2 (reverse primer) combines CLCNKB intron 3, sequence is 5'- AGCCACATGACTCTTCCTGGGCCTTTGTCTG -3'. PCR is conducted as 3-step PCR condition.

Table S5. Experimental information of ddPCR

Product	Sequence
**Forward primer; Intergenic deletion**	5’-GGAAGATTTGGGTTACTG-3’
**Reverse primer; Intergenic deletion**	5’-GGCTAATGACAATCCTGAA-3’
**Probe for Intergenic deletion**	5’-FAM-ATCTTTCCACATCCAACTCCTCTCCCT-BHQ1-3’
**Forward primer; *CLCNKA* deletion**	5’-CAGTGATCCCATCTGTGC-3’
**Reverse primer *CLCNKA* deletion**	5’-GTCCCCTAAGGTTCATTTC-3’
**Probe for *CLCNKA* deletion**	5’-FAM-CTGAATAGTAGAGCCTGTGCCACC-BHQ1-3’

Supplement Figures

Figure S2. ***CLCNKA*** **variant from maternal allele depicted in IGV and well-conserved residue of** ***CLCNKA*** **variant among the orthologs in various species**

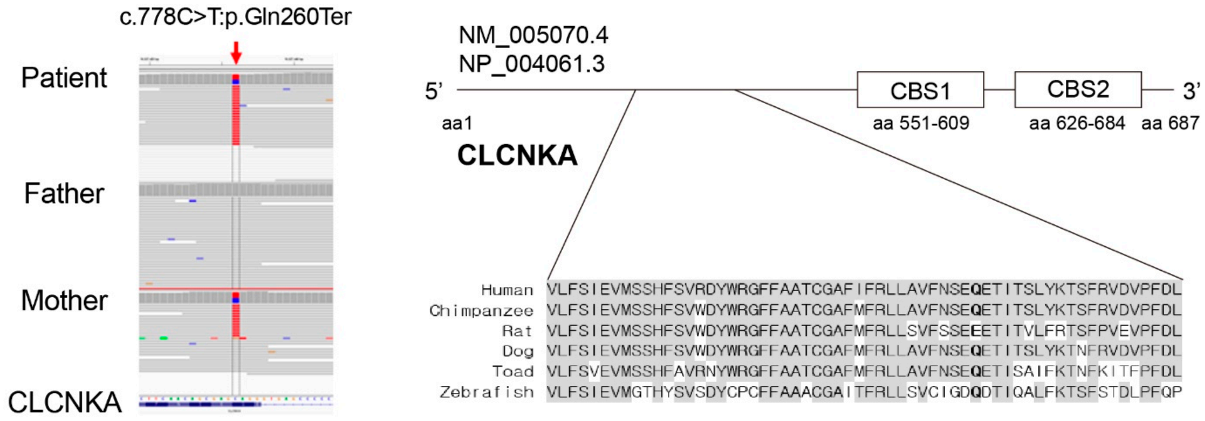

Figure S3. ***CLCNKA*** **copy number state visualized by ddPCR**

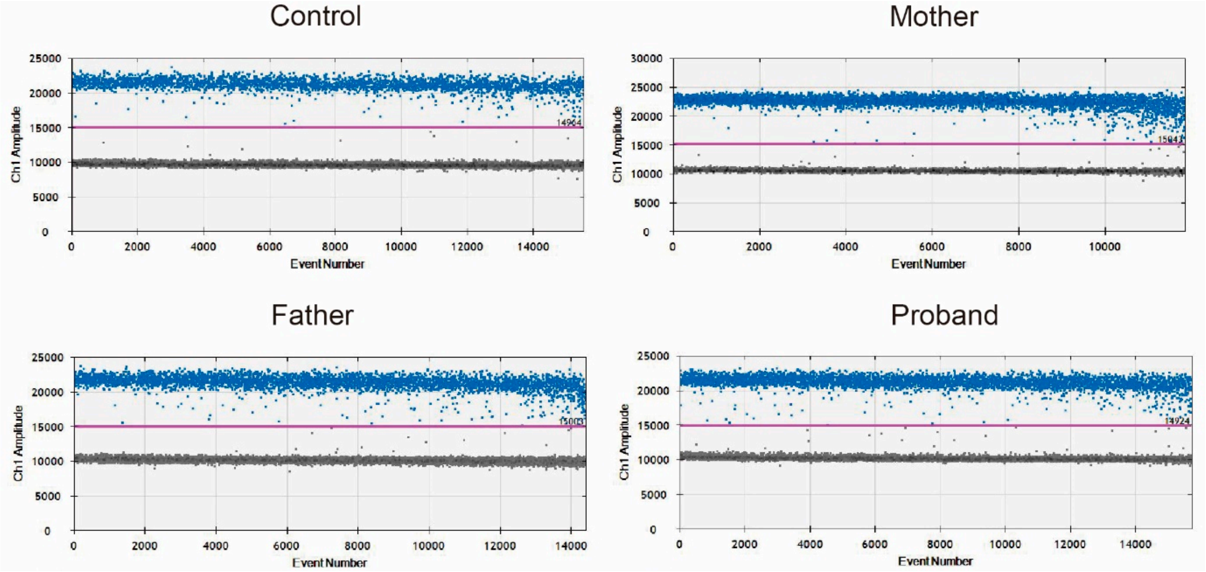

Supplement: Supplementary file 1 [file ijms-24-17077-s001.zip › ijms-2698120-supplementary.pdf]
